# Integrating social and behavior change communication into mass drug administration campaigns for neglected tropical diseases: insights and best practices from Jimma, Ethiopia

**DOI:** 10.3389/fpubh.2025.1682291

**Published:** 2025-12-03

**Authors:** Zewdie Birhanu, Morankar Sudhakar, Daba Abdissa, Gelila Abraham, Gebeyehu Bulcha, Teshome Shiferaw, Nimona Berhanu, Firanbon Teshome, Hirpa Miecha, Yohannes Kebede

**Affiliations:** 1 Department of Health, Behavior and Society, Jimma University, Jimma, Ethiopia; 2 Department of Biomedical Sciences, Jimma University, Jimma, Ethiopia; 3 Department of Health Policy and Management, Jimma University, Jimma, Ethiopia; 4 Jimma Zone Health Office, Jimma, Ethiopia; 5 School of Pharmacy, Jimma University, Jimma, Ethiopia; 6 Oromia Regional Health Bureau, Addis Ababa, Ethiopia

**Keywords:** integration, insights, best practices, SBCC, MDA campaign, Jimma, Ethiopia

## Abstract

**Background:**

Neglected tropical diseases (NTDs) remain a significant public health concern despite control efforts. Current control strategies rely heavily on mass drug administration (MDA), often overlooking complementary interventions like social and behavior change communication (SBCC). Given the complexity of behavior and its role in intervention uptake, this study explored experiences, best practices, and lessons learned from integrating a tailored SBCC approach into MDA campaigns in Jimma, Ethiopia.

**Methods:**

A descriptive qualitative study was conducted following the implementation of SBCC integrated into two MDA campaign for target NTDs (OV and STH) between June and September 2022 in Jimma, Ethiopia. Purposive sampling was used to select participants from community members and stakeholders involved in the implementation of the project. A total of four focus group discussions, five expert group discussions and 10 key informant interviews were conducted. Guided by the RE-AIM framework, data were collected through four focus group discussions, five expert group discussions, and 10 key informant interviews. The data analyses were facilitated by Atlas.ti version 7.1.5.

**Results:**

The study found that the intervention successfully reached diverse community groups through home visits, religious leaders, schools, and IEC materials, raising awareness and demand for MDA services. SBCC was integrated at key touch points, such as community registration, where early hygiene and NTD prevention education ensured informed participation. Community mobilization efforts, including local leaders and megaphones, expanded message reach, while schools and drug distributors amplified outreach and reinforced adherence. The approach was highly accepted, with stakeholders supporting its long-term sustainability and scale-up. However, challenges such as limited time, human resource constraints, and operational workload affected full integration and various adaptive strategies, helped improve implementation.

**Conclusion:**

Integrating SBCC into MDA campaigns showed strong potential in enhancing community engagement and improving treatment coverage in a context with behavioral and social barriers. While not universally required, SBCC may serve as a strategic complement in settings facing such challenges. The findings provide practical insights for guiding the context-specific application of SBCC to strengthen the effectiveness and sustainability of NTD interventions.

## 1 Background

Neglected Tropical Diseases (NTDs) represent a diverse group of illnesses primarily affecting populations in tropical regions, with over 40% of the global burden concentrated in sub-Saharan Africa (1). They are labeled “neglected” because they predominantly affect the “forgotten people” those living in rural and impoverished urban areas of low-income countries because of lack of funding and scientific effort (2). Sub-Saharan Africa faces especially severe challenges, including weak healthcare infrastructure, limited access to essential medicines, and pervasive poverty, all of which contribute to the persistence and spread of NTDs. Globally, over one billion people are affected, with Ethiopia experiencing one of the highest burdens of NTDs (3). NTDs cause significant suffering, leading to disability, disfigurement, stigma, and impairment. Several NTDs including lymphatic filariasis, onchocerciasis (OV), schistosomiasis, and trachoma, soil-transmitted helminths (STH) are known to cause significant suffering, resulting in long-term disability, disfigurement, social stigma, and impairment. They affect both physical and cognitive development, further reinforcing the cycle of poverty (4, 5). Within the sustainable development goals (SDGs), NTDs are targeted for elimination by 2030, as they affect millions and have been described as a “chronic pandemic” (6, 7).

In Ethiopia, 16 out of the 20 identified NTDs are major public health concerns, despite sustained efforts to address the issue over the years (8). Among these, OV and STH are among the 12 public health priorities by the Ethiopian Federal Ministry of Health aimed at prevention and control (8). STH is particularly prevalent, affecting over 79 million people and endemic across approximately 89% of Ethiopian districts (9). STH causes significant impact on people’s lives by contributing to malnutrition, bowel obstruction, anemia, adverse pregnancy outcomes and affect economic productivity (10, 11). The prevalence rates of STH vary widely, ranging from 18.1% to 70.3% (12–15). Onchocerciasis, another major NTD, remains a public health concern despite various control efforts. This disease is endemic in 188 districts of Ethiopia (16), particularly impacting regions such as western Oromia, the Southern Nations, Nationalities, and Peoples’ Region, Northwestern Amhara, as well as significant portions of Gambella and Benishangul-Gumuz (17, 18) (Supplementary Figure S1).

In alignment with the World Health Organization’s (WHO) strategy, the Ethiopian government has launched a comprehensive program aimed at preventing, controlling and eliminating both NTDs. This multifaceted approach includes Mass Drug Administration (MDA) campaigns, the promotion of water, sanitation, and hygiene (WaSH) practices, and public education (19). However, treatment coverage for both target NTDs remains suboptimal (20) due to various factors such as misconceptions of diseases (21, 22), logistical issues, civil unrest (23–25), poorly coordinated interventions and inadequate community engagement (26–28). Additionally, the long-term reliance on MDA delivery has raised concerns about the potential development of drug resistance and environmental threats, as these medications can contaminate ecosystems and disrupt ecological balances, even at low concentrations (29). Furthermore, specifically for STH, the program targeted specific risk groups, and in general there is limited emphasis on WaSH practices (30–32).

Both NTDs are endemic to Jimma, Ethiopia. Among household heads in peri-urban kebeles of Jimma, the overall STH prevalence was 18.1% (14). A community-based cross-sectional study in unmapped villages of the Jimma Zone reported an overall prevalence of *OV* infection at 22.5%. Additionally, 29.8% of participants exhibited onchocercal skin diseases (33). Several factors contribute to the persistence of these diseases, including climate conditions, inadequate WaSH practices, and cultural practices such as the use of rivers for irrigation and swimming activities without protective measures. Additionally, inadequate knowledge of preventive practices further exacerbates its transmission (34, 35).

To achieve sustained control and eventual elimination of NTDs, integrating SBCC strategies is particularly valuable in contexts where social and behavioral barriers hinder MDA efforts (36–39). To meet the goals outlined in the WHO 2021–2030 NTD roadmap, increasing attention to social and behavioral factors is critical particularly in areas with persistent treatment gaps or low uptake of interventions (38). SBCC plays an important role by increasing awareness, addressing misconceptions, and promoting community engagement and ownership. It employs a systematic, interactive, and research-driven communication process aimed at inducing change at individual, community, and societal levels. Its goal is to stimulate action and create an enabling environment for sustainable behavior change. By examining personal, societal, and environmental factors, SBCC can help identify key tipping points that lead to improvements in health behaviors (40).

In Ethiopia, the National Health Promotion and Communication Strategy Framework underscores the role of awareness, behavior change, and social mobilization in advancing public health goals (41). Similarly, the national NTD strategic plan (2021–2025) supports the use of community mobilization, advocacy, and behavior change communication to complement MDA campaigns. Behavior change communication is particularly recommended in settings where participation and compliance are hindered by trust deficits, misinformation, or socio-cultural barriers (3, 42). While routine MDA campaigns typically include sensitization activities, these are often limited in scope and do not fully address the broader behavioral and ecological determinants of NTDs (1, 41, 43). While evidence from some countries suggests that NTD elimination can be achieved without SBCC, its universal application may not always be necessary (44). Furthermore, prior studies in Ethiopia have shown that misconceptions about the disease and the medication negatively influence the uptake of MDA (22, 45).

In Ethiopia, the integration of SBCC into MDA campaigns has been limited (46). This shortfall represents a missed opportunity to address contextual barriers to treatment uptakes. While some studies have demonstrated the effectiveness of integrating SBCC approach into MDA campaigns in improving community knowledge, perceptions, and preventive practices related to NTDs, the overall evidence remains limited and context-specific (47, 48). Thus, SBCC should not be seen as a universal requirement for all MDA efforts but rather as a targeted strategy that may enhance outcomes where appropriate.

This study explored the integration of a tailored SBCC approach into MDA campaigns targeting NTDs in Jimma, Ethiopia. Employing a sequential mixed-methods design, we first conducted a desk review and qualitative inquiry to identify key behavioral barriers from perspectives of community and stakeholders, followed by a survey to quantify them. Based on these insights, we co-implemented a context-specific SBCC strategy alongside integrated MDA delivery. The intervention also incorporated WaSH behavior change, which is particularly critical for STH control. The findings provide practical insights into when and how SBCC can be effectively applied to enhance community engagement, strengthen participation, and support adaptive implementation. Ultimately, the study contributes evidence to inform the strategic, context-driven use of SBCC in future MDA programs to accelerate progress toward NTD elimination.

## 2 Methods

### 2.1 Study setting and design

A descriptive qualitative study approach was employed to explore insights focusing on experiences, best practices, and lessons learned from integration of tailored SBCC into MDA campaigns. The study was conducted between July and September 2022 following the end of the study. It was conducted in Jimma Zone, Ethiopia, which is located approximately 357 kilometers west of Addis Ababa, the country’s capital city. Five districts in the zone were selected based on input from experts work in the area and consideration of several criteria such as disease endemicity and focus of MDA efforts. The study was implemented in five MDA target districts for STH and OV in the Jimma zone of the Oromia region, Ethiopia. Accordingly, Gomma, Manna, Kersa, Omo Neda, and Omo Beyam districts were identified to be included in the study. These districts are identified through discussions with the zonal and regional health experts considering key criteria such as the high burden of STH and OV, the high load of microfilaria, and blackfly and poor performance of WaSH implementation status.

### 2.2 Population and sampling approach

Purposive sampling was used to select participants from community members and stakeholders involved in the implementation of the SBCC-integrated MDA. Participants were selected based on their direct involvement in and knowledge of the program to ensure they could provide relevant and informed insights. The sample included stakeholders across various levels of the health system, including primary health care units (PHCUs), district, zonal, and regional levels. Additionally, participants were purposively recruited from the community, including volunteers, beneficiaries, and community leaders. All were approached in person at their workplaces, in a comfortable and private setting, with no one else present.

### 2.3 Data collection tool and procedure

A semi-structured interview guide was developed based on the RE-AIM framework (Reach, Effectiveness, Adoption, Implementation, and Maintenance) and related literatures. This framework is widely used to evaluate public health interventions (49, 50) and was applied in this study to explore key aspects of SBCC implementation. A general discussion guide was employed to initiate conversation and elicit more in-depth information through targeted probing questions. In conjunction with the interview, the interviewer recorded field notes on nonverbal, vocal, and body language indicators. All discussions and interviews were recorded using a digital voice recorder.

The data were collected through four focus group discussions (FGDs) with different purposively selected community members from both adults and youths. Likewise, five expert group discussions (EGDs) were held with diverse groups of experts representing different positions and professional categories, including NTD and WaSH focal persons, health extension workers (HEWs), and regional and district-level program managers. Finally, 10 key informant interviews (KII) with stakeholders (community volunteers, HEWs, WaSH experts, NTD experts and PHCU leader) were held. All FGDs, EGDs, and KIIs were conducted by a team of qualitative researchers who were university instructors with master’s-level education, fluent in the local languages (Afan Oromo and Amharic), and had substantial experience in qualitative research. On average, KIIs lasted 40 min, FGDs about 1 h and 20 min, and EGDs approximately 1 h and 35 min. The team was organized into separate groups to ensure effective and context-sensitive data collection and FGDs, EGDs, and KIIs were conducted during the same data collection period.

### 2.4 Theoretical bases of the study

Our study was guided by the RE-AIM framework a widely recognized tool for enhancing the evaluation and implementation of health interventions (49, 50). RE-AIM serves as a planning and evaluation model that addresses five critical dimensions of individual and setting-level outcomes essential for program impact and sustainability. This framework was chosen for its capability to identify implementation bottlenecks and develop context-specific solutions that facilitate the translation of evidence-based interventions into practice, ultimately maximizing health impact.

**Figure 1:**
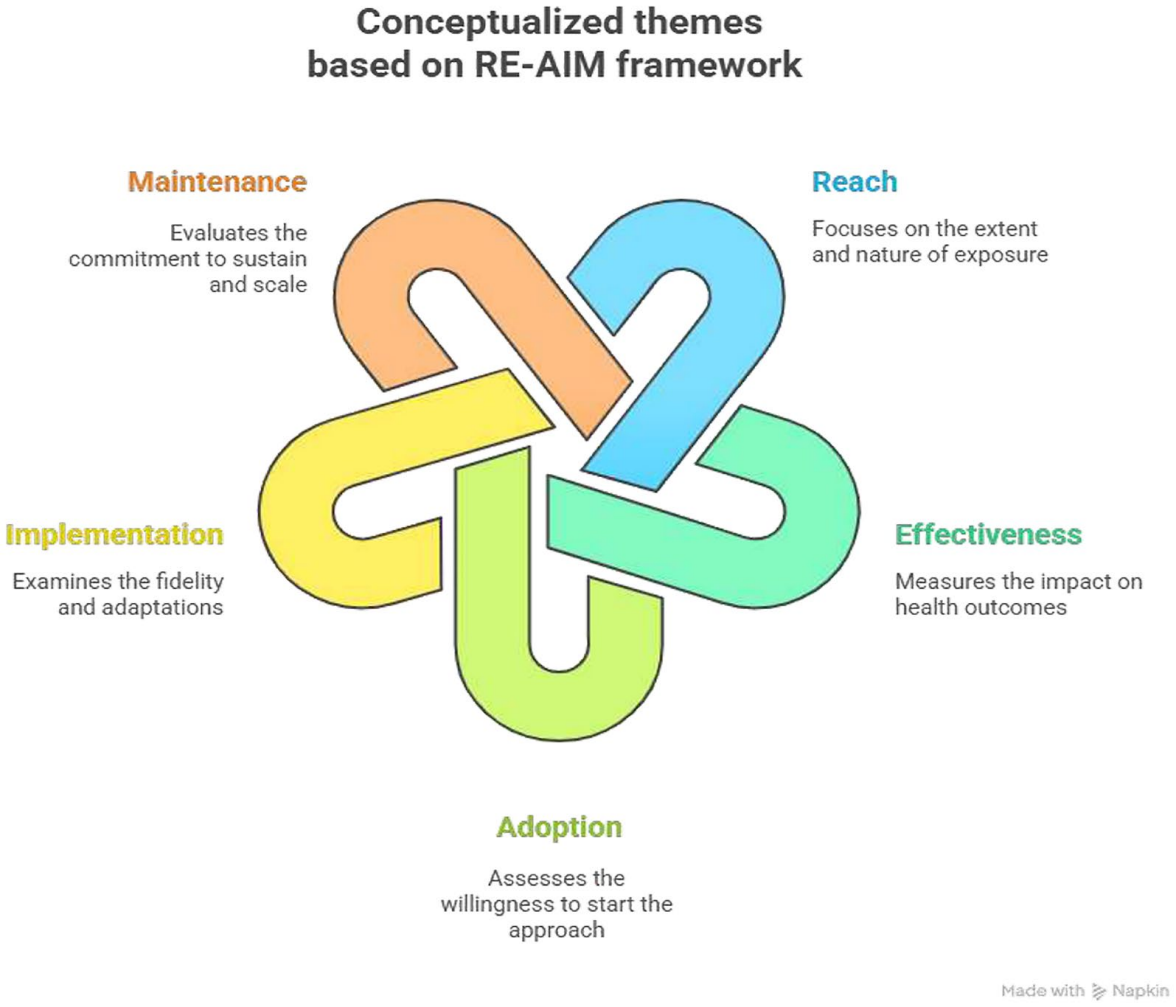
Re-AIM framework operational definitions in this study, Jimma, Ethiopia.

In this manuscript, we explored the RE-AIM constructs from participants engaged in the implementation of SBCC integrated to MDA campaign and RE-AIM was used to guide the method for “evaluation of the project.” The RE-AIM constructs were operationalized as follows (Figure 1). Additionally, acceptability is operationalized as how well the interventions are received by communities and stakeholders, including the reasons for acceptance. Findings regarding the effectiveness of the integrated SBCC interventions on targeted NTDs were partially reported in our previous publication, which focused on the impact of SBCC on improving community knowledge, perceptions, and preventive practices using a mixed-methods approach guided by the RE-AIM framework (47). In the current manuscript, we expanded the scope to include all five RE-AIM dimensions and report additional findings under the *effectiveness* domain specifically related to MDA drug uptake, community engagement and broader programmatic outcomes that were not covered in the earlier study.

### 2.5 Data analysis procedure

We employed an inductive thematic analysis approach complemented by a deductive framework guided by the RE-AIM framework. All interviews and discussions were transcribed verbatim and translated into English. Data collection was monitored daily, and saturation was determined when no new information emerged from ongoing reviews. Coding and further analyses were conducted using Atlas.ti 7.1.5. The research team independently read and coded (open coding) subsets of selected transcripts based on research questions to develop an initial code structure reflecting participants’ experiences of the phenomenon.

Subsequently, the research team, all with experience in qualitative research jointly reviewed the analysis process and emergent themes to guide the systematic coding of transcript. They shared, discussed, negotiated, and established a standardized code structure through consensus among the team. This iterative process allowed for the finalization of the analysis, emphasizing quality control, and ensuring transparency.

After identifying emergent themes, we systematically mapped them onto relevant RE-AIM constructs (Reach, Effectiveness, Adoption, Implementation, and Maintenance). Themes that did not fit within the RE-AIM framework, such as *Acceptability of SBCC* and *Best Practices*, were retained and reported separately to preserve analytical depth. This hybrid inductive-deductive approach ensured both data-driven insights and conceptual coherence. Findings are presented using major themes and subthemes, supported by representative quotes to reflect participant perspectives.

### 2.6 Data quality control

To ensure data quality, various techniques were employed. During interview and discussion sessions, facilitators summarized key points at the end of each interview and discussion, encouraging participants to provide feedback and confirm that the summaries accurately reflected their ideas. An audit trail was maintained to validate the study findings, ensuring that the results were logical and data-driven. Additionally, the entire research process, methodology, study area, and interpretation of results were described in detail to enhance transferability. Trust was established with study participants through prolonged engagement, which involved staying with the community prior to data collection, engaging in informal conversations, and clearly explaining the study’s purpose and ethical procedures. The data were reported in accordance with the Consolidated Criteria for Reporting Qualitative Research (COREQ).

### 2.7 Integration of tailored SBCC approach into MDA campaign platform

**Figure 2:**
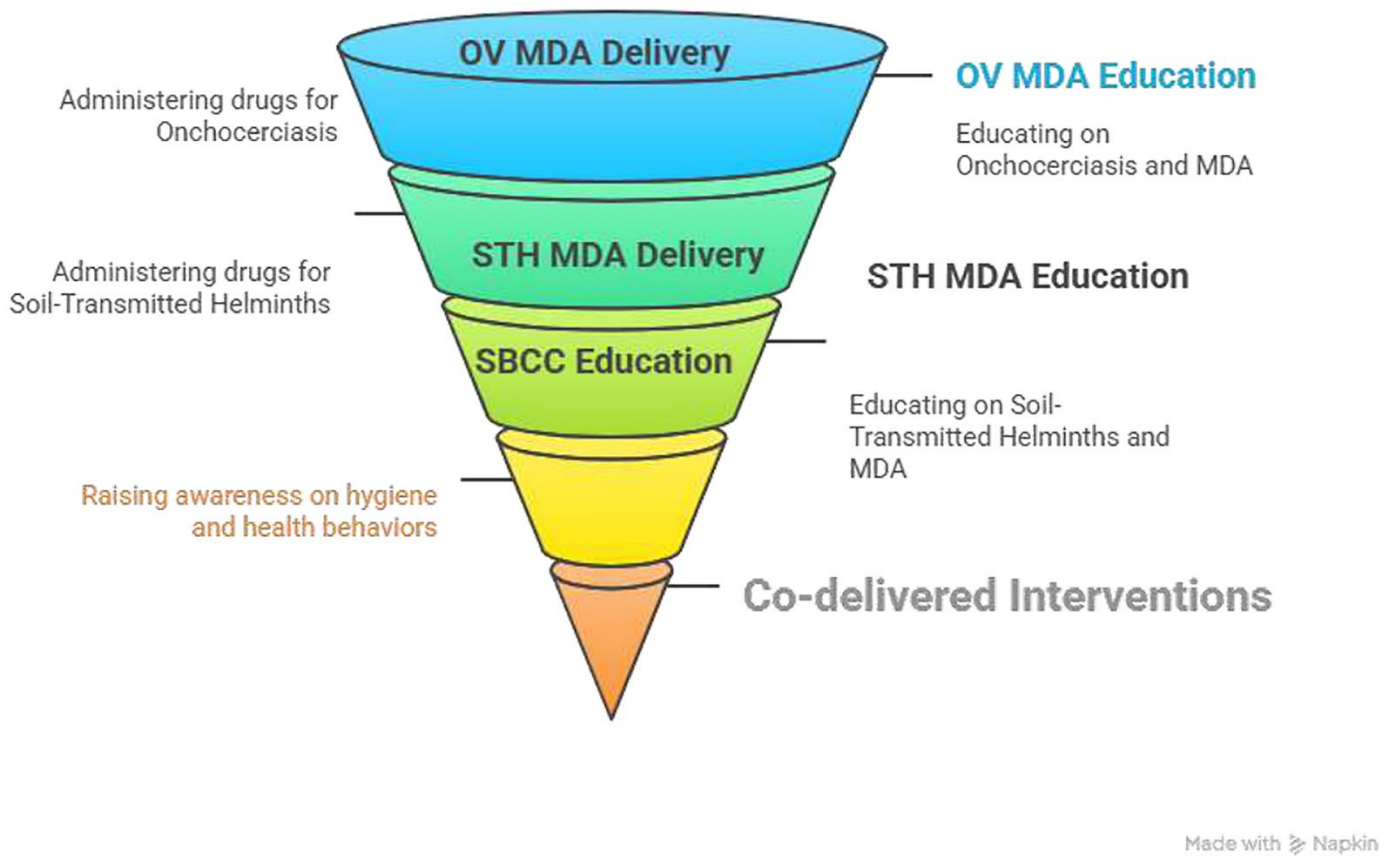
The co-delivery model including SBCC intervention of the project, Jimma, Ethiopia.

This study is a part of a larger implementation research project entitled “*evaluation of effectiveness, feasibility and acceptability of co-delivery of two MDA for OV and STH along with partial integrations of three complementary health interventions.*” The complementary interventions were water, sanitation, and hygiene promotion, promotion of COVID-19 prevention, and unvaccinated and dropout child identifications. In this study, a tailored SBCC approach was integrated into the MDA campaign platforms for two NTDs: OV and STH based on formative findings. Co-delivery in this context refers to implementing SBCC interventions that address both NTDs simultaneously using a shared strategy, communication materials, and delivery channels (Figure 2).

The SBCC interventions aimed to raise community awareness, improve preventive behaviors, increase acceptance and uptake of MDA drugs, and promote key WaSH practices. These interventions were research-informed and designed to actively engage eligible populations by delivering culturally appropriate messages about the diseases and MDA drugs thereby fostering informed and sustained behavior change.

The design of SBCC approach began with a desk review to assess the problem’s magnitude. This was followed by formative research aimed at identifying gaps and understanding the barriers and facilitators related to target NTDs, the MDA campaign, and complementary interventions, including WaSH knowledge and appropriate practices. The findings from the formative research have been published and are available elsewhere. Some of the key findings of formative assessment were high awareness but low comprehensive knowledge for both OV (48.8%) and STH (46.7%). Risk perception was low for OV (18.7%) and moderate for STH (55.2%). Preventive practices were limited (OV 47%, STH 44.4%) and linked to knowledge (34, 35).

To address the identified gaps, a strategic design was implemented. Informed by formative evidence, locally tailored SBCC messages were developed into well-designed and effective printed Information, Education, and Communication (IEC) materials, including posters, integrated brochures, IEC cards, banners, and flipcharts, all developed in alignment with the principles of IEC material development (Supplementary material S2). The messages were crafted to be culturally sensitive and acceptable, considering the cultural contexts of the target rural communities in selected districts of Jimma zone. The communication messages aimed to create understanding, motivation, and attitudinal change toward adopting healthy behaviors and acceptance of the MDA campaign platforms for target NTDs.

For each material, a creative brief was developed, followed by drafting messages. This included field visits for photos and design, completing drafts, translating, pretesting, analyzing results, and final revisions. Before production, all materials were thoroughly pre-tested with the target population to ensure clarity, relevance, and effective communication. Revisions were made based on their feedback. These materials enhance learning and reinforce messages from other channels, providing essential information while motivating community action.

### 2.8 Implementation of integrated SBCC into MDA campaign

MDA campaigns are a key strategy in the fight against NTDs, aiming to provide at-risk populations with safe and effective medications on a regular basis, without the need for individual diagnoses. In Ethiopia, MDA campaigns have played a crucial role in combating OV and STH, distributing medications to at-risk populations to reduce disease prevalence and improve public health outcomes. The drug of choice for OV is Ivermectin and target group for MDA are all person age ≥5 years in endemic areas biannually by community drug distributors in community, whereas for STH drug of choice is albendazole/mebendazole mainly for school-age children and start from 2022 started to give for reproductive age women also biannually by HEWs and support of teachers until elimination of this diseases. These campaigns have engaged health extension workers and community volunteers, with coordination from regional and district health offices.

In our study, to enhance their effectiveness, SBCC was integrated to raise awareness, dispel misconceptions, and foster community participation throughout the process. A diverse range of communication approaches was employed to maximize the reach and frequency of key messages on the target NTDs using various channels. Key strategies include the use of IEC materials, trained and oriented community volunteers, including Community Drug Distributors (CDDs) and opinion leaders, play a vital role in disseminating messages. Existing human resources, including volunteers and frontline health workers were reoriented to deliver services. The drug was distributed by HEWs and volunteers including CDDs provide supportive role. Messages were disseminated through HEWs, community volunteers, primary health care unit leaders (PHCU) health workers, local community leaders (kebele, gare, and religious leaders) and students.

Exposure opportunities for the campaign were structured at multiple levels during the community registration, pre-campaign, and intra-campaign phases. First exposure: During house-to-house visits for community registrations to identify eligible targets for the proposed MDA, trained volunteers delivered key messages about targeted diseases. They utilized flip charts to convey information on transmission and preventive measures, and distributed information cards detailing the modes of transmission, consequences, and preventive strategies for NTDs.

Second exposure: As part of community mobilization, community volunteers, frontline health workers, and community leaders provided essential health messages to community members motivating families for MDA services uptake. To support these efforts, educational materials such as brochures, posters, banners, and message cards were distributed to the community, providing an additional exposure point.

Third exposure: School-based key educational messages were delivered to students by experts from the research team and trained community volunteers. Students acted as messengers, sharing this information with their families. Key SBCC messages were disseminated in schools using banners and posters with school teacher/director (school/s located within study kebele) sensitizes the students.

Fourth SBCC contact: During the campaign event, health workers and volunteers disseminated vital information on targeted diseases and MDA services, reinforcing key messages through posters, demonstrations, and one to one counseling. The detail is described in our earlier article (47).

### 2.9 Definition of terms

#### 2.9.1 Kebele

The smallest administrative unit in Ethiopia, functioning as a local government structure under a district (woreda). A kebele typically consists of several villages and is responsible for community-level administration, service delivery, and mobilization.

#### 2.9.2 Gare

A sub-division within a kebele, usually comprising a cluster of households (often 25–30), formed to facilitate community organization, health service delivery, and local development activities.

#### 2.9.3 Community and stakeholder engagement

Community and stakeholders were engaged in the research process, as well as the co-design and co-implementation of the strategy. The community played a pivotal role in every stage of the study process. A formative consultative study was conducted to gain a nuanced understanding of who to engage and how to do so effectively. During this phase, we consulted with community representatives, potential partners, and stakeholders at various levels (community, district, zonal, and regional) to identify the challenges, barriers, facilitators, enablers and opportunities for integration of campaign package. This collaborative approach enabled us to pinpoint the most effective strategies for co-delivery. Furthermore, community members including community leaders, community volunteers from selected districts actively co-designed the intervention strategy through a participatory workshop with key stakeholders on target NTDs (OV and STH). They made significant contributions to various aspects of the project, including registration, validation of IEC materials, intervention design, community mobilization and education. A key strategy for research uptake was the close engagement of key stakeholders and policymakers in all aspects of the study. In this study, the study’s underlying research questions were framed from the perspectives of health system challenges, ensuring that input from health personnel aligned with the needs and interests of both local and national health systems. Key stakeholders were actively participated throughout the research process ensuring their insights and needs were incorporated. The detailed integration strategy was developed collaboratively through participatory training workshops with stakeholders.

#### 2.9.4 Capacity building and training procedures

Training was provided for campaign coordinators, implementers, community volunteers, PHCU workers, and health extension workers for implementing the intervention. A training manual was developed by adapting existing resources, including manuals and guidelines, with key modifications such as detailed content specifications, training duration, the number of trainees, etc. based on the findings from formative assessment and stakeholders’ feedback. The material was developed in English but translated to the local language (Afan Oromo) for use by frontline health workers and the campaign implementation team. This initiative was designed to ensure effective delivery of the campaign through a well-prepared workforce.

The training procedures began by introducing the overall content of each chapter. Clear objectives for each session were outlined, followed by the formation of buzz groups as needed. Overall brainstorming questions were posed to represent the main contents of each session. Participants were given approximately 5 minutes to note their responses to the questions on the space provided in the manual or on a flipchart, if available. Scenarios were then presented to describe each session of the chapter, helping to initiate discussions among learners. They were asked to share their feelings and experiences regarding the stated scenario before and after the sessions. Finally, a summary of the key contents of each session and chapter was provided. Resources such as markers, flipcharts, notebooks, and other materials were utilized during the training.

### 2.10 Reflexivity statement

The research team, with expertise in public health and social science, collaboratively designed and conducted this study, remaining mindful of potential biases. Through reflexive journaling, diverse data sources (KIIs, FGDs, expert discussions), and collaborative analysis, the team strove for transparency and a nuanced understanding of the data, ensuring participants’ voices remained central to the findings. Furthermore, the analysis process involved joint reviews with research team members of emerging themes, with systematic coding guided by a consensus-driven, iterative approach to ensure quality control and transparency in the final analysis.

## 3 Results

### 3.1 Participants’ demographics

This study involved 10 KIIs with various stakeholders who played key roles in the implementation. These participants included two HEWs, one PHCU deputy director, one from zonal health office, one each of district level WaSH and NTD focal persons and four community volunteers. Similarly, focus group discussions were conducted with four distinct groups from community members: married individuals age ranges from 30 to 56 and youths age range from 16 to 26 years (Supplementary material S3). Finally, five EGDs were conducted with groups of two to four participants, involving various experts at PHCU (3), district program managers with NTD and WaSH focal persons (1), and regional program managers (one discussion) (2–4 participants per group) (Table 1).

**Table 1:** Background characteristics of the expert group discussion, Jimma, Ethiopia.

S/N	ID	Sex	Role
1	P1	F	HEW
	P2	F	HEW
	P3	F	HEW
2	P1	F	HEW
	P2	F	HEW
3	P1	M	Regional health bureau CDC director
	P2	M	National NTD team leader
	P3	M	Regional NTD team leader
	P4	M	STH-Schistosomiasis focal person at Regional health bureau
4	P1	M	District level NTD focal
	P2	F	District level WaSH focal
5	P1	F	HEW
	P2	F	HEW

Key: HEW, health extension worker; CDC, communicable disease control; P, participant.

### 3.2 Integration experiences of SBCC approach into MDA campaign

**Figure 3:**
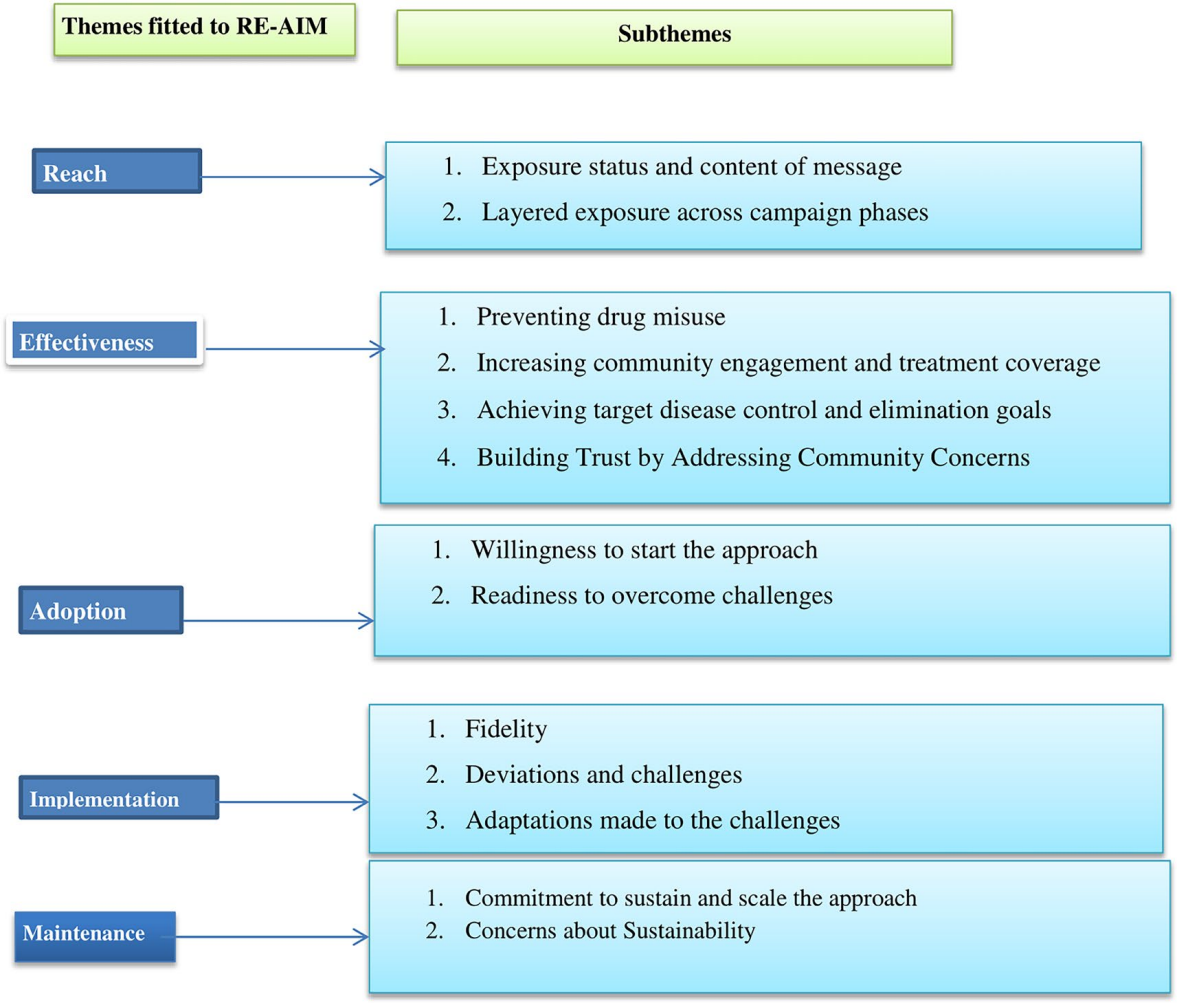
Themes and sub-themes by RE-AIM dimension, Jimma, Ethiopia.

The experiences of integrating SBCC into the MDA campaign were analyzed through the RE-AIM framework to assess the reach, effectiveness, adoption, implementation, and maintenance of the intervention (Figure 3).

This explorative analysis indicated that integration of tailored SBCC approach into the MDA campaign effectively reached diverse community members through multi-layered strategies, including home visits, community mobilization efforts, and school-based outreach. The following section presents detailed of the findings.

### 3.3 Reach

The survey revealed, 88.8% of the respondents reported exposure to SBCC information. From this, 164 (21.1%) of them were exposed once and the rest exposed to SBCC information more than one. The SBCC integrated into MDA campaign achieved wide community exposure to SBCC messages through multiple communication channels and campaign phases.

#### 3.3.1 Reach status and content of message

This study found that the intervention successfully reached diverse community groups through home visits, religious leaders, schools, and IEC materials, raising awareness and demand for MDA services. Participants consistently described the dissemination of messages related to hygiene, sanitation, disease prevention, and the importance of drug administration.

“…They [HEW] have also taught us about latrine utilization and hygiene by going home to home…Honestly speaking, HEWs are teaching us.” (P2, married women, FGD)

“….During the current campaign, we reached almost all eligible individuals…” (KII, HEW)

Community discussions and social interactions further amplified SBCC messages beyond formal outreach efforts, embedding health-related conversations in everyday life.

“It is impossible to say that people didn’t hear the message. It’s being talked about even at coffee ceremonies.” (FGD, P2, Male Youth)

#### 3.3.2 Expansion beyond the target community

The campaign’s impact extended beyond the intended areas, sparking interest among residents from neighboring communities. Many individuals outside the target population actively sought information and requested drugs after hearing about their benefits.

“…there were many people from other kebeles who heard about it and come to our office to ask us about it.” (KII, HEWs)

#### 3.3.3 Layered exposure across campaign phases

The campaign strategically embedded SBCC at multiple stages, increasing the likelihood of repeated exposure and message reinforcement (Table 2) and Supplementary material S4.

**Table 2:** Layered SBCC exposure across campaign phases.

Campaign phase	SBCC strategies/activities	Illustrative quotes
Community registration	First touch point for SBCC dissemination. Volunteers provided household-level health messages. Used IEC/visual aids to illustrate disease impact and prevention.	*“We have already been giving orientation to the public home to home… we convinced them that oncho drug is used to prevent such condition.” (KII, Volunteer)* *“We used health learning materials to educate the community about hygiene and latrine use.” (FGD, Volunteer Youth)*
Community mobilization	HEWs engaged Gare leaders/CDDs to mobilize households. Public announcements (megaphones). Gatherings used for broader health education (disease transmission, hygiene, nutrition). Kebele and religious leaders reinforced credibility. IEC materials distributed.	*“The HEWs conducted community mobilization in many ways… they called Gares or CDD members a day before.” (KII, PHCU Deputy Head)* *“When we do community mobilization, nothing is done without involving the kebele structure.” (KII, PHCU Deputy Head)* *“We often use religious leaders to transmit our message via the mosque.” (EGD, HEW)* *“…when mobilize the community, they would gather at the same place and prodiing health education…”(P1, EGD, HEW)*
School-based dissemination	Students educated at school relayed messages to households. Approach ensured wide reach and accuracy. Recognized as more effective than some traditional mobilization routes.	*“Students never forget what they have been told… They convey the exact message.” (EGD, HEW)* *“The students come from everybody’s home… ensuring full coverage.” (HEW, EGD)* *“Messages delivered through students have greater reach than those through CDDs [community drug distributors].” (HEW, EGD)*
Drug distribution point	HEWs/volunteers delivered tailored health messages during drug administration. Clarified dosage, symptoms, and transmission. Used posters and visual aids. Encouraged peer mobilization by early attendees.	*“When we deliver the drugs, we say… taking the drugs alone is not useful unless they keep their hygiene.” (KII, Volunteer)* *“Health professionals explained why some take 4 pills and others 3.” (FGD, Youth)* *“There was a large paper posted on the site, and HEWs explained the pictures.” (FGD, Married Women)* *“We have also used those people returning from the campaign sites to inform or mobilize the latecomers.” (KII, PHCU Deputy Head)*

### 3.4 Acceptability of integrating SBCC into MDA campaigns

#### 3.4.1 Acceptability status

This analysis indicated that the integration of SBCC into MDA campaigns was widely accepted by both beneficiaries and stakeholders. Respondents appreciated the program’s approach, with one stakeholder noting, *“The integrated delivery of services providing health education alongside ivermectin or Albendazole is very much appreciated, and we often receive positive feedback from the public” (KII, PHCU deputy head).*

Health Extension Workers and WaSH focal persons also expressed strong support, with one HEW stating, *“The community highly accepted the current approach” and a WaSH focal person emphasizing, “All components, including community registration, pre-campaign, intra-campaign, and post-campaign activities, are acceptable.” (KII, HEW, KII, WaSH Focal).*

There was initial community skepticism due to limited awareness, which shifted to acceptance once the intervention’s relevance was understood, highlighting the essential role of SBCC in building trust and perceived value.

“Hummm… the community was found saying, “what is its relevance for us” before they get some level of awareness about it. However, after we went through the community and create awareness, it was found good. They accepted because they believed it must be useful for them.” (KII, Volunteer)

#### 3.4.2 Reasons for acceptance

This study identified key reasons for acceptance, highlighting the importance of trusted communication, education from health professionals, and support in the successful integration of SBCC into MDA campaigns (Table 3).

**Table 3:** Reasons for acceptance of approach with explanation and representative quote, Jimma.

Reasons for acceptance	Narrative and quotation
Increased awareness creation through credible sources	Community acceptance was enhanced by increased awareness through trusted sources. One participant shared, “*The community accepts the information transmitted by HEWs. They have a high network [high acceptance and relation] with the community” (FGD, 46-year-old male).* *“The people liked education to increase their awareness…, because in the previous time, there was no awareness creation. People only take the drugs given to them, our fathers know nothing about what’s going on” (KII, Volunteer)*
Perceived value of education from credible sources in enhancing acceptance	The community highly valued receiving advice from health professionals. A youth participant stated, *“A piece of health advice is more healing than the service itself… People are happy when they get advice about the disease, its consequences, and how to prevent it” (P7, Youth, FGD).* *“…. People easily understood and accepts it once you explain for them. So, we, the health workers, HEW and I[volunteer] myself tried to address all this people’s concerns” (KII, PHCU deputy head)*
Supportive Supervision and Resources	Supportive supervision and the provision of resources were crucial for acceptance. A HEW emphasized, “*In the recent campaign, all things are good from Jimma University and the woreda health office. I am very interested in the supplies, including IEC materials and supervision” (P2, EGD, HEW).* *“…We have been following this campaign on daily basis…” (KII, PHCU deputy head)*

### 3.5 Adoption dimension

The findings indicated an inclination among stakeholders to adopt the integrated SBCC into MDA approach. Most participants recognized its potential to enhance NTD control efforts by promoting collaboration, addressing behavioral barriers, and generating evidence that could inform long-term health system improvements.

#### 3.5.1 Willingness to start the approach

Stakeholders at multiple levels including zonal health officials and frontline health workers expressed strong enthusiasm toward initiating the integrated approach. They viewed the initiative not only as a technical advancement but also as a strategic opportunity to strengthen health systems and improve community outcomes.

*“As Jimma Zone Health Office, we’ve been delivering interventions for various NTDs for years… but this collaboration with Jimma University is a great opportunity. This evidence-based project will help identify challenges and strengths, and lessons learned will be adapted to future NTD campaigns…. the findings from this project could also be a lesson and can be adopted to the health systems in the country.” (KII, Jimma Zonal Office)*

Health Extension Workers echoed this sentiment, highlighting their readiness and interest in co-delivering integrated messages and treatment.

*“…We are highly interested in it [co-delivery] and ready…” (P3, EGD, HEW)*

However, while the willingness to adopt was high, some participants emphasized that sustainable implementation would require addressing key logistical and resource-related enablers.

*“… To enhance its effectiveness and adoption in the future, it’s important to address adequate manpower. …So, our probability to adopt it in the future depends on whether the manpower issue is resolved” (KII, PHCU Deputy Director)*

### 3.6 Effectiveness dimension

The integration of the SBCC approach into the MDA campaign was perceived as highly effective, with treatment coverage reaching 89.5% of the eligible population (*n* = 3,733) for OV and 84.1% (*n* = 2,610) for STH. Stakeholders and community members highlighted that SBCC contributed to improved MDA coverage, prevention of drug misuse, and stronger community engagement. Several perceived benefits of SBCC integration are summarized below.

#### 3.6.1 Preventing drug misuse

Participants reported a significant improvement in drug use compliance during the recent MDA campaign compared to previous years. In earlier campaigns, drug misuse was reportedly common often driven by a lack of understanding about the purpose and benefits of the drugs. However, this trend has shifted following intensified awareness efforts through SBCC activities.

Participants explained that increased understanding among community members helped address misconceptions and encouraged proper drug intake.

*“…The first one is due to a lack of understanding that people need to use the drug for other purposes…But now, the people are able to understand its benefits…the people are ready to participate when told for.” (P3, FGD, CDD Volunteer and P5, FGD, Youths)*

This enhanced awareness led to stronger community participation and accountability during the campaign.

*“….There was no drug misused during the recent campaign…” (P1, youth, FGD)*

Moreover, the campaign design and delivery were perceived to be more organized and transparent, reducing opportunities for malpractice. A Health Extension Worker highlighted the following:

*“….solved a number of issues such as impartiality and malpractice.” (P1, HEW, EGD)*

Improved supervision and the use of directly observed treatment (DOT) also contributed to this change, as expressed by a community volunteer:

*“…There wasn’t taking the drugs home, everyone has swallowed the drugs in front of us”. …since the current approach allows the public to directly swallow it in front of the health workers, it has big benefit…” (KII, Volunteer)*

#### 3.6.2 Increasing community engagement and treatment coverage

Participants noted improved community acceptance of MDA drugs over time, attributing this to increased awareness and home-based education by HEWs. Previously resistant individuals are now more motivated and willingly participate in the campaigns.

*“…previously there were individuals who never wanted to take the drugs. Nowadays, the HEWs are able to move home to home, teach those individuals, and convince them to take it…” (P4, FGD, Youth)*

Additionally, community members were reported to respond positively to peer influence and word-of-mouth messaging from early participants and community members who had already attended the campaign.

*“The community comes out after they get heard of the messages from those who attend the campaign earlier and from other ways…” (P2, HEW, EGD)*

Motivation to participate appeared strong, with respondents describing how people actively came forward for treatment after understanding its preventive value.

*“….But everyone won’t miss it. There was no problem. No one was careless since they know the benefit of this onko drug in preventing the possible disease. People come here more motivated to receive.” (P1, FGD, Youth)*

Health workers also confirmed that drug coverage had improved, reaching nearly all eligible individuals.

*“…drugs have adequately reached out everyone eligible in the community….” (EGD, P2, HEW)*

Furthermore, participants observed a notable increase in overall community knowledge and proactive behavior related to MDA services.

*“…Now the community has got a lot of awareness about the benefit of the drugs. They would even come to kebele level to seek the service.” (P1, FGD, Male Youth)*

#### 3.6.3 Building trust by addressing community concerns

While initial concerns such as skepticism, confusion, and mistrust were observed during the registration phase, volunteers played a crucial role in addressing them.

*“… During the registration time, I tell the community about the campaign… they complain saying, ‘If you will give the drugs to gares [CDD] as usual, why would you register us? There is no reason to register us!’ However, we provided advice that things have changed… In doing so, we are able to convince them and do the registration…” (KII, Volunteer)*

Another recurring concern was the safety of taking multiple drugs at once. Volunteers helped ease such fears by emphasizing the role of the government in ensuring drug safety and clearly communicating the health benefits of the medications.

*“People concerned about the number due to combination of the drugs. However, we assured them that we won’t give them harmful things that are provided by the government, rather we educate them that it a good thing that benefit their health…” (KII, Volunteer)*

#### 3.6.4 Achieving target disease control and elimination goals

Stakeholders highlighted the approach’s potential to contribute significantly to the control and eventual elimination of targeted diseases.

*“…In general, the current approach is very fruitful and helps to eliminate diseases….” (P3, EGD, HEW)*

### 3.7 Perceived sustainability

Stakeholders showed strong commitment to sustaining and institutionalizing the integrated SBCC approach within MDA. The perceived community benefit, improved drug uptake, and positive feedback from the public served as key motivators for both continued implementation and scale-up to other areas. However, while many participants demonstrated optimism, a few raised concerns regarding long-term sustainability without continued external support.

#### 3.7.1 Commitment to sustain and scale the approach

The findings indicate a shared commitment among stakeholders to both sustain and expand the integrated SBCC approach within MDA. Despite challenges such as increased workload, participants emphasized the importance of continuing the co-delivery model due to its perceived benefits for the community.

*“…we feel this approach should be strengthened and continued in the future…” (P7, FGD, Youth)*

There was also enthusiasm to scale the approach to other districts, reflecting both institutional and community-level support.

*“… It would also be interesting to expand it to other Gandas and districts. We have been receiving feedback from the community to continue with the current approach…” (KII, HEW)*

In addition, community demand for the drugs underscores the long-term viability of the intervention and integration of SBCC messaging into local health behavior.

*“Even, the experience be drawn from it and must be expanded to areas where it wasn’t implemented yet, because, when you see the benefits the community gained from it, you will be convinced to continue implementing it…” (HEW, P1, EGD)*

#### 3.7.2 Concerns about sustainability

Despite the widespread support, a contrasting view emerged that highlighted potential risks to sustainability, particularly in the absence of supervision and training support.

*“… As is implemented right now, I don’t think it would continue especially if those people who provided us the training stopped supervising. Those who provide us the training have been closely visiting us and observing how we deliver the activities while were in the field…” (KII, Volunteer)*

### 3.8 Implementation status

The implementation of the tailored SBCC approach integrated in to MDA campaign largely aligned with planned strategies, yet challenges related to human resources, leadership engagement, and community behavior influenced fidelity and effectiveness. Stakeholders identified key challenges and adaptation strategies to enhance implementation success.

Perceived Fidelity: Alignment with Plans and Deviations.

Participants acknowledged that the intervention was implemented in close alignment with the initial plan, ensuring key activities were carried out as intended. *“We carried out all the activities as we planned…. We have not faced this much problem! We have successfully accomplished an interesting job” (EGD, P1, HEW).*

However, not all stakeholders shared the same experience. Some reported implementation gaps caused by contextual challenges, especially human resource constraints. The shortage of trained personnel made it difficult to carry out all components of the plan effectively, particularly when multiple tasks of registration, measurement, drug administration, and health education had to be conducted simultaneously.

*“…In connection with the shortage of health professionals, we have faced a lot of challenges. Look, the activities require registration, measuring, giving the medicines or providing health education on the other hand causes challenges. We didn’t complete the task as per our plan.” (KII, PHCU Deputy Head)*

Despite successes, stakeholders mentioned several deviations that affected fidelity across the different SBCC exposure points, during the campaign event, community mobilization, and community registration (Table 4; Supplementary material S5).

**Table 4:** Deviations, challenges, and adaptation strategies during implementation.

Implementation stage	Challenges (illustrative quotes)	Adaptation strategies (illustrative quotes)
Campaign events	Limited human resources and time constraints. *“The main challenge we faced is a shortage of teams and it is tiresome.” (HEW, EGD)* *“…there was the challenge of human resources during the intra-campaign period.” (KII, HEW).* Short campaign duration. *“…The other challenge was the shortage of campaign duration. As a result, we were unable to provide health education to all the attendants.” (KII, HEW)* Community resistance to health education. *“Since our people come from rural areas and have little awareness, they will not tolerate waiting.” (HEWs, EGD)* *“They will not wait when you stop giving the medicine to provide health education. They return home angrily…” (KII, HEW)*	Extending campaign duration. *“…we have been re-conducting the campaign in many settings… we were forced to extend the days of the campaign.” (KII, PHCU Deputy Head)* Group management and flexible scheduling. *“…We divided the public into groups… sometimes conducted health education sessions after administering drugs.” (HEWs, EGD)* *“…I have tried to do it [education] in parallel.” (KII, HEW)* Personal dedication. *“…we were forced to pass the entire day in the field… we did not feel any form of hunger because we are serving the public.” (KII, Volunteer)*
Community mobilization	Inadequate coverage. *“We cannot say our community mobilization is perfect… we did not actually go to the village.” (KII, PHCU Deputy Head)* Limited leadership engagement. *“The Ganda leader was busy and did not fully engage in the campaign.” (KII, HEW)* *“The kebele administration did not fully participate in disseminating messages…” (KII, NTD focal)* Delayed message dissemination. *“There is disparity among Gares… health workers suffered from repeated visits.” (FGD, Youth)*	Flexible communication approaches. *“…we used our phone calls. In areas where mobilization failed, we moved to gares and engaged residents directly.” (KII, HEW)* *“By sending messages to students if the gares failed… we were also successful.” (HEW, EGD)* Utilizing past campaign experiences. *“…we were transmitting the information using all chances we get, such as community meetings.” (HEW, EGD)*
Community registration	Resource-intensive process. *“Registering the community by moving home to home is very difficult… This will need many human resources and takes time.” (HEW, EGD)*	

### 3.9 Best practices and recommendations suggested for effective SBCC integration into MDA

Stakeholders proposed practical recommendations to enhance the effectiveness, scalability, and sustainability of SBCC integration within MDA campaigns. Four key areas were highlighted.

- Pre-campaign preparation was considered critical, with calls for stronger planning, early involvement of Ganda leaders, and prioritization of health education before the start of drug distribution to ensure communities are well informed.
- Community engagement and mobilization should be strengthened through integrated multi-channel approaches and strong leadership involvement to foster trust and compliance.
- Capacity building and human resource management emerged as another priority. Stakeholders emphasized the need for additional staff at campaign posts, enhanced recognition and motivation for volunteers, and the engagement of educated youth in registration to improve efficiency and data quality.
- Strong supportive supervision was highlighted as essential in addressing challenges related to CDD motivation and performance through training, incentives, or replacement where necessary.
- Finally, ensuring system sustainability and trust was seen as vital for scale-up. This included building multi-stakeholder acceptance across communities, government levels, and partners (Table 5).

**Table 5:** Best practices and recommendations for SBCC integration into MDA campaigns.

Subtheme	Best practices/recommendations	Illustrative quotes
Pre-campaign activities	Develop a logical plan and ensure thorough preparation. Prioritize health education before campaign launch.	*“…strong pre-campaign preparation including discussion with Ganda structures for community mobilization.” (KII, WaSH Focal)* *“…Our plan has to be logical. We need to avoid placing the full workload on individuals…” (EGD, WaSH Focal)* *“…The best time for health education is during community mobilization before starting the campaign…” (HEW, EGD)*
Strengthening community engagement and mobilization	Integrate multi-channel mobilization (IEC, registration events, school-based outreach). Active participation of HEWs in household visits. Tailored IEC material dissemination. Engage local leadership structures.	*“Community registration is a good opportunity… School is also an enabling factor…” (HEW, EGD)* *“…HEWs have been persuading everyone… going down to home level to come and receive the services.” (FGD, Youth)* *“…Printed health information materials were full of important points…” (EGD, HEW)* *“…If the Ganda leader is involved in the campaign, we can surely achieve 100%.” (EGD, HEW)*
Capacity building and human resource management	Expand human resources and logistics support at campaign posts. Engage and motivate volunteers. Improve registration with educated volunteers. Good supportive supervision.	*“…Each campaign post needs additional human resource who provide health education and register beneficiaries.” (EGD, HEW)* *“…Volunteers have been serving the community… it would be beneficial if some motivation was provided.” (KII, HEW)* *“…Use committed students after providing training and improving their awareness.” (KII, PHCU Deputy Head)* *“…It would be implemented appropriately since their eyes are closely observing the activities….” (KII, Volunteer)*
Enhancing system sustainability and trust	Build multi-stakeholder trust and acceptance. Formalize partner engagement and integration. Address challenges among CDDs (motivation, retraining, replacement).	*“…The approach needs to be accepted and trusted by the community, service providers, implementers, and government system…” (EGD, Regional Health Bureau)* *“…If donors integrate resources including budget, drugs, and training… it is good for sustainability.” (EGD, NTD focal person)* *“…It is important to understand why CDDs resisted… and provide incentives or replace underperforming CDDs.” (EGD, Regional NTD team leader)*

## 4 Discussion

This study explored experiences, best practices, and lessons learned from integrating a tailored social and behavior change communication (SBCC) approach into two combined mass drug administration (MDA) campaigns targeting onchocerciasis (OV) and soil transmitted helminthes(STH) in Ethiopia. Typically, these MDA campaigns are implemented separately and biannually as vertical programs with limited coordination or communication between them. In this study, both MDAs were integrated and supported by a context-specific SBCC strategy guided by the RE-AIM framework (49, 50). Integrating SBCC into combined MDA campaigns is particularly important when delivering potential drug combinations, as it can enhance community awareness, address concerns about treatment safety, foster acceptance, and promote knowledge and preventive behaviors in targeted populations.

Findings across the RE-AIM dimensions were encouraging, with the tailored SBCC approach receiving strong acceptance and support from stakeholders for potential sustainability and scale-up. Stakeholders at multiple levels expressed willingness to adopt and sustain the approach in contexts where it addresses identified barriers. Key touch points including community registration, mobilization activities, and the MDA event itself proved effective for embedding SBCC interventions, providing repeated and reinforced delivery of essential messages. The use of well-designed printed IEC materials further expanded message reach within target communities. Notably, message spillover into neighboring kebeles demonstrated the potential for peer-to-peer diffusion, consistent with the WHO’s 2021–2030 NTD roadmap emphasis on community-centered, cross-cutting strategies (38). These findings suggest that leveraging multiple, context-appropriate opportunities is critical to maximizing the impact of SBCC efforts.

The implementation of the tailored SBCC approach within the MDA campaign largely followed the planned strategies; however, full integration faced several challenges. These included shortages of human resources, limited time allocated for SBCC activities, leadership gaps, community impatience, and the resource-intensive demands of community registration. To address these issues, implementation teams adopted adaptive strategies such as flexible communication methods, improved group management, and adaptable scheduling to maintain the intervention’s integrity. These findings are consistent with existing literature highlighting the importance of tailoring interventions to local contexts to enhance effectiveness (38, 51). Additionally, some community drug distributors (CDDs) showed reluctance to engage fully, underscoring the need to foster ongoing dialogue, clarify roles, and involve CDDs in inclusive planning processes. Such pragmatic adaptations reflect the RE-AIM framework’s focus on context-responsive implementation rather than strict protocol adherence.

Consistent with previous findings (42, 52) which highlight communication as a central tool in NTD elimination strategies, SBCC is vital for controlling NTDs by improving health knowledge and promoting preventive behaviors such as hygiene, sanitation, and acceptance of MDA campaigns. Our findings further demonstrate that integrating tailored SBCC into MDA campaigns enhances awareness, addresses misconceptions, and fosters community engagement, thereby strengthening community ownership and contributing to the effectiveness and sustainability of NTD control efforts. Stakeholders widely perceived this approach as beneficial, highlighting its potential to contribute meaningfully to disease control, elimination, and prevention of drug misuse. Our study suggests that SBCC helps address fears of adverse events and improves risk perception, with effective health communication serving as a key driver of behavior change. Given the critical role of human behavior in NTD control (53), it is important to recognize the complexity of behavior and the multitude of factors that influence it.

Our findings align with existing evidence showing that SBCC interventions are particularly important in rural communities (54–56), where they promote accurate knowledge and encourage preventive behaviors related to NTDs. Other study has documented that MDA initiatives often encounter resistance stemming from lack of awareness or misconceptions about program goals (57). Moreover, effective disease control requires addressing underlying social and environmental determinants not merely delivering treatment echoing the principle that “treating people without changing what makes them sick” is unsustainable (58).

Several factors contributed to the positive outcomes observed in this study, notably the active involvement of communities and stakeholders throughout the research process. The co-design and co-implementation of the SBCC approach fostered a sense of shared responsibility and relevance, which likely enhanced both uptake and sustainability. Participatory approaches are widely recognized for strengthening the effectiveness of health interventions by building trust, encouraging collaboration, and promoting mutual accountability (59, 60). Community engagement, in particular, plays a pivotal role in cultivating local ownership and empowerment, which are essential for long-term sustainability (61). These findings reinforce the importance of involving communities in health behavior change efforts, as informed and actively engaged populations are more likely to support, adopt, and maintain interventions over time (62, 63). Knowledge also plays a crucial role in shaping health-related behaviors; greater understanding of disease risks and consequences supports preventive practices among at-risk populations and promotes adherence to control programs (64). Nonetheless, it is important to note that evidence from some countries indicates that NTD elimination can be achieved without SBCC, suggesting that while highly beneficial, its universal application may not always be essential (44).

This study highlighted several best practices for enhancing the reach, effectiveness, adoption, and sustainability of NTD interventions. These include co-designing tailored communication strategies and using diverse channels to expand reach and influence behavior. Strengthening campaign fidelity may involve allocating resources for additional educators or trained volunteers and offering refresher training and modest incentives to sustain CDD motivation. Designating stand-alone education days before drug distribution can help reduce crowd impatience and promote informed participation. Lastly, active stakeholder collaboration particularly through joint micro-planning fosters shared ownership, minimizes miscommunication, and ensures alignment with campaign objectives.

While this study was conducted in Jimma, Ethiopia, the tailored SBCC-integrated MDA approach is potentially adaptable to other endemic regions with similar socio-cultural and health system contexts. However, variations in infrastructure, community dynamics, and disease burden may affect implementation and outcomes. Future research should assess the approach’s effectiveness across diverse settings and examine its long-term impact and cost-effectiveness to inform scalable strategies for advancing NTD control and elimination. This study was strengthened by the use of a robust implementation science framework (RE-AIM), which enhanced the credibility, depth, and applicability of the findings. The active engagement of stakeholders and community members throughout the design and implementation phases ensured contextual relevance, fostered trust, and promoted shared ownership critical elements for the success and sustainability of behavior change interventions. However, several limitations should be acknowledged. Some resistance from community drug distributors, potentially due to perceived shifts in roles and expectations human resource constraints, limited campaign duration and inconsistent leadership participation, may have influenced the fidelity of implementation. Additionally, this study did not assess cost-effectiveness, and as it represents a component of a larger project, not all RE-AIM dimensions were fully explored. Furthermore, cost effectiveness analysis, unintended consequences and the broader implications of co-creation were not examined in detail. Despite these limitations, this study provides compelling evidence to support the integration of tailored, community-driven SBCC strategies into MDA campaigns. It underscores the potential for such approaches to be scaled and adapted across similar settings and offers important directions for future implementation research, including comprehensive evaluation of long-term impact and economic feasibility.

## 5 Conclusion

This study demonstrated that integrating a tailored SBCC approach into an MDA campaign yielded promising results, successfully reaching a broad audience through diverse communication channels. It enhanced community participation, improved treatment coverage, and supported efforts toward sustained disease control in a context where social and behavioral barriers were present.

While these findings underscore the potential value of SBCC in strengthening NTD control programs, they also highlight the importance of context-specific application. SBCC should be considered as a strategic complement in settings where community engagement challenges, misconceptions, or treatment refusals are prevalent not as a universally required component of every MDA campaign.

Future program design should focus on identifying when and where SBCC is most appropriate. Strengthening local community capacity, engaging stakeholders early and meaningfully in planning and implementation, and developing clear operational strategies will be essential to enhance both the fidelity and effectiveness of SBCC supported MDAs where needed.

## Data Availability

The raw data supporting the conclusions of this article will be made available by the authors, without undue reservation.
