# Supplementary material for: Integrating social and behavior change communication into mass drug administration campaigns for neglected tropical diseases: insights and best practices from Jimma, Ethiopia
Source: Front Public Health. 2025 Dec 3;13:1682291. doi: 10.3389/fpubh.2025.1682291 (PMC12708591; doi:10.3389/fpubh.2025.1682291)
Supplement: Supplementary file 1 [file Data_Sheet_1.docx]

**Supplementary Files**

**Supplementary figure 1: Figure on Onchocerciasis endemic districts in Ethiopia.**


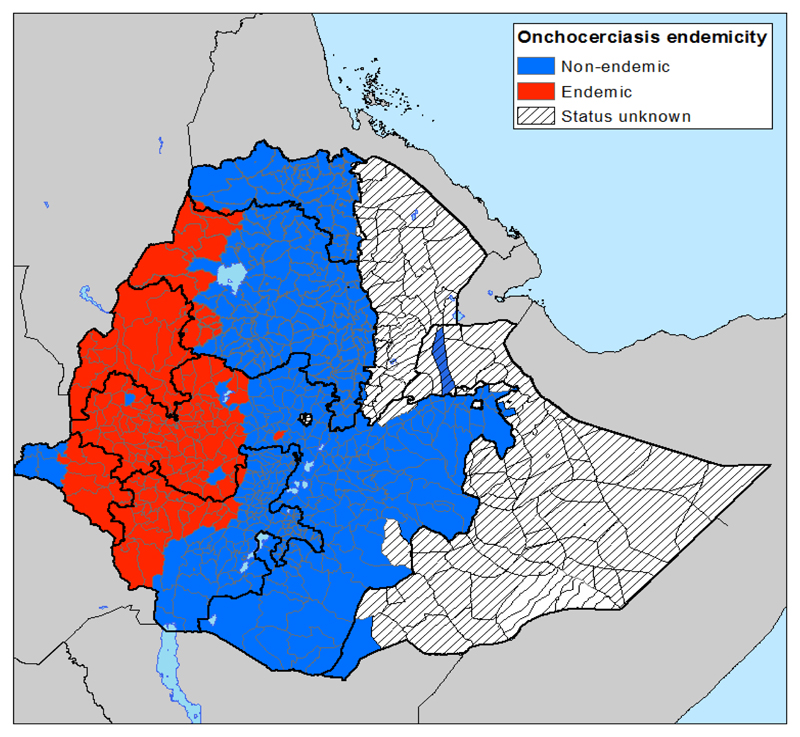


**Supplementary table 1: SBCC materials produced with their purpose, Jimma, Ethiopia.**

| **Materials** | **Who and where to use** | **How to use** | **Main contents** |
| --- | --- | --- | --- |
| Brochure | Communities, Volunteers, HEWs, other MDA team | Distributed to the general community who can read during campaign and through school children | Provides detailed information on target NTDs its definition, cause, prevention, symptoms, mode of transmission, consequences, and encourage to take MDA and other prevention and control methods in local language |
| Flipchart | Registrants, HEWs, MDA team, Health Workers | Use a flip chart to conduct family dialogue and one to one client education during house to house registrations and onsite | Detailed messages on target NTDs and its prevention practice including MDA in local language |
| Information Card | Volunteers, HEWs, MDA team, Health Workers | Distributed to all households during service delivery and community registration | Containing key messages such as consequences, mode of transmission and preventive measures of targeted NTDs in easily understandable format Has also information on date of campaign for MDA |
| Poster | Health facilities, schools, and public gatherings. | Mounted at public gathering places and campaign sites where people can read and understand | Focused on target NTDs and their prevention, in bold visuals, eye-catching graphics designed to capture attention from a distance |
| Banners | Research and MDA teams | For announcements of campaign events | Key messages on campaign day, services to be given to eligible individuals and WaSH practices |

**Supplementary table 2: Background characteristics of the KII and FGD participants, Jimma, Ethiopia.**

| **Number of KII** | **Position/role** | **Educational status** | **Experience** | |
| --- | --- | --- | --- | --- |
| 1 | Jimma zonal health office |  |  | |
| 2 | NTD focal person | BSc health officer | 5 years | |
| 3 | District WaSH Focal | Diploma | 9 years | |
| 4 | Volunteer | Grade 6 |  | |
| 5 | Volunteer | Grade 10 |  | |
| 6 | PHCU deputy director | BSc health officer |  | |
| 7 | Volunteer | Grade 12 |  | |
| 8 | Volunteer | Grade 10 |  | |
| 9 | HEW | Level 4 |  | |
| 10 | HEW | Level 4 | 9 years | |
| **Background characteristics of the FGD participants, Jimma, Ethiopia** | | | | |
| Group | Participants | Number of participants | Age range | Education |
| 1 | Male married | 6 | 33 to 56 | 2 to 10 |
| 2 | School age boys | 8 | 17 to 26 | From grade 8 to 10 |
| 3 | School age girls | 6 | 16 to 20 | From 6 to 8 |
| 4 | Women married | 7 | 30 to 43 | No formal education to grade 10 |

**Supplementary Text 1: Layered exposure across campaign phases.**

The campaign strategically embedded SBCC at multiple stages, increasing the likelihood of repeated exposure and message reinforcement.

- **During community registration:** Community registration served as the first major touch point for SBCC dissemination. Volunteers and health workers used this stage to deliver targeted health messages while enrolling households in the campaign.

*"We have already been giving orientation to the public home to home regarding oncho drugs while conducting registration… So, we have convinced them that oncho drug is used to prevent such condition..."(KII, volunteer)*

Additionally, registration was used to inform residents about the upcoming campaign, reinforcing early awareness and engagement.

*"…We communicated the community during the registration that the drugs will be distributed for them very soon…" (KII, Volunteer)*

To enhance understanding and engagement, health extension workers and volunteers employed visual aids and IEC materials. These materials helped illustrate disease impact and promoted positive health behaviors.

*"We have been using materials to show the bad effects of the disease to the public.* *So, we have convinced them that oncho drug is used to prevent such condition…" (KII, Volunteer)*

*"We used health learning materials to educate the community about hygiene and latrine use." (FGD, P4, Volunteer Youth)*

- **During Community Mobilization:** The second key opportunity for SBCC exposure occurred during the community mobilization phase, where stakeholders employed diverse communication strategies to engage residents and maximize campaign reach.

Health Extension Workers utilized multiple mobilization methods, such as contacting Gare leaders or CDDs in advance to prepare the community.

*"The HEWs conducted community mobilization in many ways. For instance, they called Gares or CDD members a day before the campaign to mobilize the community." (KII, PHCU Deputy Head)*

Public announcements via megaphones were also common to gather community members and raise awareness. *"We used megaphones to announce and mobilize the community." (EGD, P1, HEW)*

Large public gatherings during mobilization provided valuable opportunities for health education on a range of topics including disease transmission, personal and child hygiene, and nutrition*. “…when mobilize the community, they would gather at the same place and proving health education for the public gathering is so interesting. We used to educate them many things. For instance, we educate them about ways transmission of the works, about how onko occurs, how to wash children and keep their hygiene, about child nutrition, etc. It’s possible to create awareness of the public on many issues using the opportunity” (P1, EGD, HEW)*

The involvement of local leadership, particularly kebele and religious leaders, was critical in mobilizing the community and reinforcing SBCC messages. Their participation enhanced the credibility and acceptance of the campaign.

*"When we do community mobilization, nothing is done without involving the kebele structure. If they resist you, you can’t move forward. That’s why we first notify the kebele leader and zone leaders—they are respected and feared by the public." (KII, PHCU Deputy Head)*

*"We often use religious leaders to transmit our message to the community via the mosque. They are willing to help us deliver messages during Jumu’ah [Friday prayer]."* *(EGD, HEW, P2)*

Participants perceived the campaign as inclusive and far-reaching, noting that no major groups were left behind.

*"The HEWs, with the support of the kebele structure, mobilized the public. No one was left behind. It properly reached everyone." (FGD, P1, Youth)*

In addition, IEC materials were distributed to literate community members, who were encouraged to further educate their families, thereby extending the campaign’s reach.*" As much as possible, we distributed IEC materials to those who could read and instructed them to educate their family members about what they learned." (HEW, EGD, P1)*

- **School-Based Dissemination:** Schools served as powerful SBCC dissemination hubs. The students to convey campaign messages to their families an approach praised for its accuracy and reach:

*“Students never forget what they have been told… They convey the exact message.” (EGD, HEW, P2)*

Unlike earlier mobilization methods, student involvement ensured that every household received campaign messages, overcoming gaps left by other mobilization efforts.

*"The students come from everybody’s home; the gares can’t reach out all places with the messages, students accurately relay messages to their parents and neighbors. When we educate them at school, they take the message directly to their families, ensuring full coverage." (HEW, P2, EGD)*

Participants further illustrated the approach's effectiveness by referencing other public health campaigns, such as immunization, where student-based communication had outperformed other mobilization routes.

*“Messages delivered through students have greater reach than those through CDDs. For example, in immunization campaigns, we observed a notable difference many more people brought their children when they heard the message from students rather than from Gares. Students don’t forget what they’re told, unlike many Gares.” (HEW, P2, EGD)*

- **At the Point of Drug Distribution:** The final exposure opportunity came during drug administration. Here, HEWs and volunteers delivered tailored health messages and clarified drug-related concerns:

*“when we deliver the drugs, we been saying to the community taking the drugs alone is not useful, unless they keep their hygiene of personal, children, keep the sanitation of homes, toilet, is good. We provide this education just before administering the drugs…” (KII, Volunteer)*

*“Health professionals explained why some take 4 pills and others take 3, along with symptoms and transmission of the disease.” (FGD, Youth, P7)*

*"We educated the community about the benefits of the drugs, the DOT [Directly Observed Therapy] strategy, and encouraged them to mobilize their families and neighbors to take the drugs." (KII, HEW)*

Beneficiaries also mentioned that HEWs and volunteers used materials to explain key messages during the campaign.

*"... There was a large paper posted on the campaign site, and HEWs and the volunteers were telling us about the pictures on that paper." (FGD, P4, married women, P6, women, 40 years)*

In addition, peer mobilization was employed by encouraging early attendees to inform those who had not yet participated:

*“We have also used those people returning from the campaign sites to inform or mobilize the latecomers or people who didn’t hear it.” (KII, PHCU deputy head)*

**Supplementary text 2: Deviations, challenges** and **adaptation strategies during implementation**

**A. During campaign events**

- **Limited Human Resources and Time Constraints**

A lack of sufficient personnel placed pressure on existing teams, hindering their ability to deliver health education alongside drug distribution:

*“The main challenge we faced is a shortage of teams and it is tiresome” (P2, HEW, EGD)*

*“Regarding giving health education during campaign implementation, it was not done as our plan because of lack of enough human resource…”* (EGD, HEW, P1)

Furthermore, the CDDs were not interested in the program by feeling their role is overtaken by other person which leads to challenge in human resourse.

*“….CDDs were not interested. They didn’t want the drugs distributed by health workers and as a result, they didn’t engage in the campaign. In some posts where there are no volunteers so health care providers, we had been distributing the drugs alone. So, there was the challenge of human resources during the intra-campaign period” (KII,HEW)*

- **Short Campaign Duration**

The limited timeframe restricted the ability to provide thorough health education to all participants.*“…The other challenge was the shortage of campaign duration. As a result, we were unable to provide health education to all the attendants.”* (KII, HEW)

- Community Resistance to Health Education

Some community members were reluctant to wait for educational sessions and preferred to receive treatment quickly.

*“Since our people come from rural areas and have little awareness, they won’t tolerate waiting. They start rushing, saying, ‘I have a burning issue, I couldn’t stay.’”* (P1, EGD, HEWs)

*“They will not wait when you stop giving the medicine to provide health education. They return home angrily.”* (KII, HEW)

**B. During Community Mobilization**

- **Inadequate Community Mobilization Coverage**

Some teams were unable to conduct thorough grassroots mobilization:

*“We can't say our community mobilization is perfect because we didn't actually go to the village and mobilize the public.” (KII, PHCU Deputy Head)*

- **Limited Leadership Engagement**

Community leaders were not always fully engaged, affecting campaign reach and message dissemination:

*“The Ganda leader was busy and didn’t fully engage in the campaign. If the Ganda leader is involved, we can surely achieve 100%.” (KII, HEW)*

*“The kebele administration did not fully participate in disseminating messages before campaign time.” (KII, NTD focal)*

- **Delayed Message Dissemination**

Some gare leaders did not deliver campaign messages in a timely manner, requiring repeated outreach:

*“There is disparity among Gares in disseminating the messages. As a result, health workers suffered from repeated visits to the site.” (P7, FGD, Youth)*

**C. During Community Registration**

- **Resource-Intensive Process**

Household registration required significant time and human resources, placing additional strain on implementation teams.

*“Registering the community by moving home to home is very difficult… This will need many human resources and takes time”(P2, HEW, EGD)*

*“Community registration is the most difficult part of the co-administration process. It is even difficult to get the people to their homes”(P3, HEW, EGD)*

**3.6.3 Adaptation strategies to enhance SBCC reach**

To overcome these challenges, stakeholders implemented various strategies to maximize community engagement and ensure the effectiveness of SBCC efforts.

- **Extending Campaign Duration**

One key adaptation was extending the duration of the campaign to accommodate community members who were initially missed due to absence or workload-related delays. Limited staffing necessitated prolonged implementation timelines beyond what was originally planned.

*“…Since it was extremely challenging to try to manage one team…, we have been re-conducting the campaign in many settings. … there were individuals who didn’t attend… engaged in daily routine… Thus, we were forced to extend the days of the campaign.” (KII, PHCU Deputy Head)*

- **Utilizing past campaign experiences**: Recognizing potential resistance, teams maximized opportunities to disseminate messages.

*“…We were thinking that the community may resist taking the drugs due to their previous experience that they were taking the drug at their home. Due to this fear, we were transmitting the information using all chances we get, such as community meetings…”* (EGD, P1, HEW)

- **Flexible communication approaches**

While community mobilization and outreach through gare leaders were part of the planned communication plan, implementation challenges such as delayed or incomplete message dissemination by some local leaders required immediate adjustments. In response, HEWs adapted their communication approach by directly engaging households within the gares and supplementing outreach with phone calls and school-based messaging.

*“Since we are familiar with the community, we used our phone calls. In areas where community mobilization failed, we moved to gares and engaged residents directly.”* (KII, HEW)

*“By sending messages to students if the gares failed to deliver the messages, by coming close to the public… We were also successful in this regard.”* (P1, HEW, EGD)

- **Group management and flexible scheduling**: To manage large crowds and address time constraints, health workers adapted by delivering health education alongside drug distribution.

*“…We divided the public into groups and kept them according to their turn, and sometimes conducted health education sessions after administering drugs.”* (P2, EGD, HEWs)

*“…I have tried to do it [education] in parallel.”* (KII, HEW)

- **Personal dedication and extended field presence**

This adaptability extended beyond communication techniques to include personal commitment and work ethic. Several frontline workers reported skipping lunch breaks and staying in the field for extended hours to ensure the community was fully served:

*“…we were forced to pass the entire day in the field even at lunch since many people attended, we have to serve them. We didn’t go home at lunch time. We use take away lunch and ate at the sites. We didn’t feel any form of hunger because we are serving the public” (KII, Volunteer)*
